# Comparative serum proteome expression of the steroid-induced femoral head osteonecrosis in adults

**DOI:** 10.3892/etm.2014.2069

**Published:** 2014-11-12

**Authors:** YUXIAN CHEN, CHUN ZENG, HUA ZENG, RONGKAI ZHANG, ZHIQIANG YE, BANGRONG XING, KUNHUA HU, MINGTAO LI, DAO ZHANG CAI

**Affiliations:** 1 Department of Joint Surgery and Orthopaedic Trauma, The Third Affiliated Hospital, Sun Yat-Sen University, Guangzhou, Guangdong 510630, P.R. China; 2 Department of Joint Surgery, The Third Affiliated Hospital, Southern Medical University, Guangzhou, Guangdong 510630, P.R. China; 3 Department of Joint Surgery and Orthopaedic Trauma, The Fifth Affiliated Hospital, Sun Yat-Sen University, Zhuhai, Guangdong 510630, P.R. China; 4 Proteomics Laboratory, Zhongshan Medical College, Sun Yat-Sen University, Guangzhou, Guangdong 510630, P.R. China

**Keywords:** steroid-induced osteonecrosis of the femoral head, proteome, serum, biomarker, α-2-macroglobulin

## Abstract

Steroid-induced osteonecrosis of the femoral head (SONFH) is a disabling, aseptic and ischemic disease that develops following steroid therapy. The pathogenesis of SONFH is unclear, so the early diagnosis and treatment for this disease is yet to be established. The purpose of the present study was to identify potential biomarkers for SONFH. The differential expression of serum proteins from patients with SONFH and healthy volunteers was analyzed by the proteomics method. The protein samples were labeled and subjected to isoelectric focusing and two-dimensional gel electrophoresis. The resultant protein spots were matched and quantified by an imaging analysis system. The differentially-expressed protein spots were subjected to in-gel trypsin digestion followed by matrix-assisted laser desorption ionization time-of-flight mass spectrometry. Significantly lower levels of complement component 3 (C3), C4, inter-α-trypsin inhibitor heavy chain H4 and α-2-macroglobulin were found in the serum of patients with SONFH. These proteins are reported to be actively involved in intravascular coagulation, apoptosis and reactive oxygen species imbalance, indicating that multiple pathological reactions occur in SONFH and these proteins may serve as potential biomarkers for the diagnosis of SONFH.

## Introduction

The steroid-induced osteonecrosis of the femoral head (SONFH) is a devastating, irreversible and disabling disease developing following steroid therapy (1). The functions of the hip joint are markedly impaired when the femoral head collapses. Glucocorticoid (GC) therapy was the most common cause of ONFH (2). The onset of SONFH is within several months following the administration of steroids. Regardless of continuous steroid administration, no expansion of the necrotic area was found, and recurrence was not identified. Due to ischemia, patients have no symptoms when SONFH occurs. No pain is noted until the femoral head collapses. Approximately 5–25% of patients using GC develop SONFH in the legs. Notably, in China 53.5% of patients with severe acute respiratory syndrome prescribed GC developed femoral head osteonecrosis, and the SONFH comprises approximately half of non-traumatic femoral head necrosis. In total, >50% of patients with femoral head osteonecrosis admitted to The Third Affiliated Hospital (Sun Yat-Sen University, Guangzhou, Guangdong, China) between January 2000 and January 2010 had a history of using GC, the majority of whom were young males. At present, patients with SONFH already exceed 10 million in China. However, the pathogenesis of this disease remains largely unknown, so the prevention and treatment have yet to be established.

With the progress of systemic biology, proteomic approaches provides a valuable tool to study the whole protein content of a biological sample in one set of experiments. One of the reliable technologies is two-dimensional (2-D) electrophoresis or 2-D difference gel electrophoresis (DIGE) analysis coupled with mass spectrometry identification (3,4). This technology has recently been employed to screen potential bioactive molecules underlying the pathogenesis of arthritis and osteopenia, and it is reported that the protein levels in serum, joint fluid and bone tissue correlate to the severity of rheumatoid arthritis (5), the autoimmune response in ankylosing spondylitis in animal models (6), the loss of cartilage integrity in osteoarthritis (7) and estrogen loss-induced osteoporosis (8). However, the changes detected in the serum protein profile of patients with SONFH have not yet been reported.

The purpose of the present study is to find potential biomarkers of the SONFH by using proteomic technology to analyze serum protein profiles in patients with SONFH and the healthy control group.

## Materials and methods

### Patients

The study was approved by the Institutional Review Board of The Third Affiliated Hospital, and informed consent was obtained from each patient. A total of 12 patients, including five females and seven males, fulfilled the inclusion and exclusion criteria. Inclusion criteria included receiving a single short-course of corticosteroid medication within the three years prior to presentation, ONFH identified by typical magnetic resonance imaging (MRI) findings, stage 1 or 2 by the Ficat classification system and no previous treatment of the femoral head (9). The exclusion criteria for sampling were the history or evidence of metabolic bone diseases, including hyper- or hypoparathyroidism, Paget’s disease, renal osteodystrophy and the presence of cancers with bone metastasis. In total, 12 healthy volunteers, including five females and seven males, were enrolled in the control group. Peripheral venous blood (5 ml) was drawn from each patient in the Outpatient Department or in the procedure room prior to general anesthesia for total hip replacement. The blood samples were processed to collect serum and stored at −80°C until analysis.

### Serum sample preparation

Blood samples (3 ml) from each subject were collected in a drying tube early in the morning and allowed to clot for 1 h at room temperature. The samples were centrifuged at 2,500 × g for 15 min at 4°C. The supernatant was dispensed into 0.5 ml aliquots and stored at −80°C until use. All the serum samples were processed according to a standard protocol.

### Depletion of high abundance proteins and quantification

All the samples were thawed at room temperature. High abundance proteins, such as albumin and immunoglobulin G (IgG), were depleted with the ProteoPrep^®^ Blue Albumin & IgG Depletion kit (Sigma-Aldrich, Inc., St. Louis, MO, USA) as per the manufacturer’s instructions. Subsequently, samples were further cleaned with the Clean-up kit (GE Healthcare, Piscataway, NJ, USA) according to the manufacturer’s instructions. Proteins were quantified using 2-D Quant kit (GE Healthcare). The protein concentrations of samples were adjusted to 8 mg/ml and verified by SDS-PAGE and Coomassie blue staining.

### Fluorescent labeling

To limit experimental variation and ensure accurate in-gel matching, all the samples were labeled with fluorescence. The CyDye DIGE Fluor (minimal dye) labeling kit (GE Healthcare) was used for tagging, following the manufacturer’s instructions. Cyanine 2 (Cy2) was used for the internal standard sample that was generated by mixing together an aliquot of samples from the patient and control groups. Cy3 and Cy5 were used to label samples from the control and patient groups, respectively. The labeling reaction was carried out in the dark on ice.

### 2D-DIGE and in-gel trypsin digestion

All the labeled samples were subjected to 2D-DIGE with 24 cm immobilized pH gradient (IPG) strips (pH 4–7) (GE Healthcare) for the first dimensional isoelectric focusing and subsequently the second dimensional SDS-PAGE with 12% polyacrylamide gels. The 2D-DIGE was run in triplicate for each sample to reduce the gel-to-gel variation. The gels were scanned using Typhoon 9410 Variable Mode Imager (GE Healthcare) at the excitation/emission wavelengths specific for each CyDyes immediately. Subsequent to scanning, all the gels were stained with Deep Purple and stored for subsequent mass spectrometric identification. Images were then processed with DeCyder Differential in Gel Analysis V6.0 software (GE Healthcare) to identify changes in spot fluorescence intensities. Proteins were considered differentially expressed if the abundance showed >1.5-fold change between the patient and the control groups with P<0.05 using one-way analysis of variance. Disparate points were excised using the Ettan spot handing workstation (GE Healthcare), then destained and subjected to in-gel trypsin digestion. Peptides were extracted for subsequent mass spectrometry.

### Matrix-assisted laser desorption ionization time-of-flight mass spectrometry (MALDI-TOF-MS/MS) and protein identification

Following trypsin digestion, protein identification was carried out on the Ettan MALDI-TOF mass spectrometer (GE Healthcare). The trypsin auto-digestion peaks (m/z 842.509 and 2211.104 Da) were used for internal calibration. Each spectrum corresponded to the sum of 200 acquisitions for each of eight laser pulses, in which the threshold signal/noise exceeded a set value. The resulting data were then analyzed with Mascot search engine (Matrix Science, London, UK) for protein identification and compared to the National Center for Biotechnology Information (10) and Swiss-Prot (11) protein databases. The following keywords were used in the search: Trypsin digestion, *Homo sapiens*, 1–100 kDa protein mass, 100 ppm peptide tolerance.

### Western blot analysis

Serum protein samples prepared as described above were diluted 1:25 in Laemmli buffer and resolved by 10% SDS-PAGE (Invitrogen Life Technologies, Carlsbad, CA, USA). The separated proteins were transferred to polyvinylidene fluoride. The membranes were blocked with 5% skimmed dry milk in Tris-buffered saline containing 0.05% Tween 20 (TBST) for 1 h at room temperature and incubated with antibodies overnight at 4°C. Subsequent to washing two times in TBST, the membranes were incubated with corresponding horseradish peroxidase-conjugated secondary antibody for 1 h at room temperature. Protein bands were detected using ECL Plus (Forevergen Bioscience Co., Ltd., Guangzhou, China) and the images were acquired by the Imaging System (Gel Doc XR System, Bio-Rad, Hercules, CA, USA).

### ELISA

ELISA assays using a microtiter plate assay were performed individually on the samples in each group chosen randomly and matched across the groups. Primary and secondary antibodies were obtained from Santa Cruz Biotechnology, Inc. (Santa Cruz, CA, USA). A standard curve was generated by four-parameter curve-fitting using SoftmaxPro V 1.11 software, (Molecular Devices Corp., Sunnyvale, CA, USA).

### Statistical analysis

The data analyses were performed by SPSS 15.0 (SPSS, Inc., Chicago, IL, USA). Statistical significance of the differences was determined using the Student’s t-test, and the Tukey method was performed to correct multiple comparisons. All the values were reported as the mean ± standard deviation, and P<0.05 was considered to indicate a statistically significant difference.

## Results

### Patients

In total, 12 patients with SONFH (age, 32.3±2.3 years; range, 25–40 years) and 12 healthy volunteers (age, 33±2.3 years; range, 28–41 years) were enrolled. There was no significant difference in the age (P=0.827) between the SONFH and healthy volunteer groups. All the patients were diagnosed with ONFH by MRI. The patients classified as stage 1 or 2 by the Ficat classification system were enrolled in the study. The medical records show that all of them had received glucocorticoid therapy due to arthralgia. The mean steroid dose in equivalent milligrams of prednisone was 850 mg (range, 290–3300 mg). The mean time from administration of steroids to the development of hip symptoms was 16.6 months (range, 6–33 months), but none of them fulfilled the diagnosis criteria of rheumatoid arthritis.

### 2-D DIGE analysis of differential protein expression

**Figure 1:**
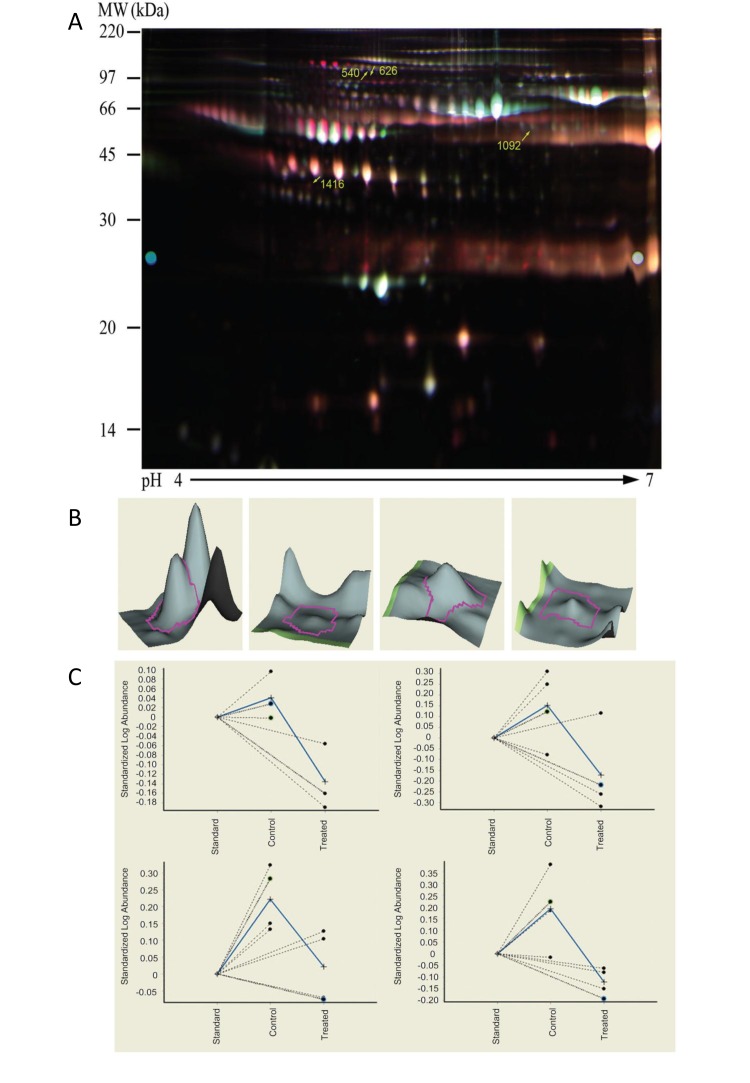
Proteomic profiling of ONFH sera revealed by 2-D DIGE analysis. Proteins extracted from the sera of the SONFH and control groups were differentially labeled with Cy3 and Cy5, respectively. An internal standard containing proteins from a mixture of sera from the two groups was labeled with Cy2 and loaded in all the gels. immobilized pH gradient strips (pH 4–7) were used for isoelectric focusing prior to standard SDS-PAGE (12.5% polyacrylamide). (A) Representative 2-D DIGE overlay image, with control in green and ONFH in red. Spots marked with a number indicate proteins whose abundance was significantly different between ONFH and control, within a 95% confidence level. (B) 3-D views of four spots that exhibit lower abundance in patients with SONFH. (C) The expression levels of the four proteins shown on 2-D DIGE were calculated with the DeCyder analysis software and presented as standardized log abundance. ONFH, osteonecrosis of the femoral head; SONFH, steroid-induced ONFH; Cy, cyanine; 2-D DIGE, two-dimensional difference gel electrophoresis; mw, molecular weight.

2-D DIGE was performed to analyze differential protein expressions as previously described (12). Approximately 1,600 protein spots were detected across all four gels by the DeCyder image analysis software (GE Healthcare). Fig. 1A shows the superimposed images in pseudocolor from Cy3- and Cy5-labelled sera samples and the positions of the spots corresponding to the proteins revealed by 2-D DIGE analysis. Fig. 1B presents the differences in protein expression by the DeCyder 3-D spot simulations. Four protein spots with >1.4-fold decrease in abundance between the patient and control groups were identified by computer-assisted comparative analysis (P<0.05, Student’s t-test; Table I). To further confirm the change of these four protein spots, two preparative gels loaded with 500 μg protein from each extract were run in parallel and followed by Deep Purple staining. The decrease of the protein abundance was consistent with the result of CyDye labeled images as analyzed by DeCyder software. Four proteins were revealed to be downregulated in the sera of patients with SONFH.

### Protein identification

**Figure 2:**
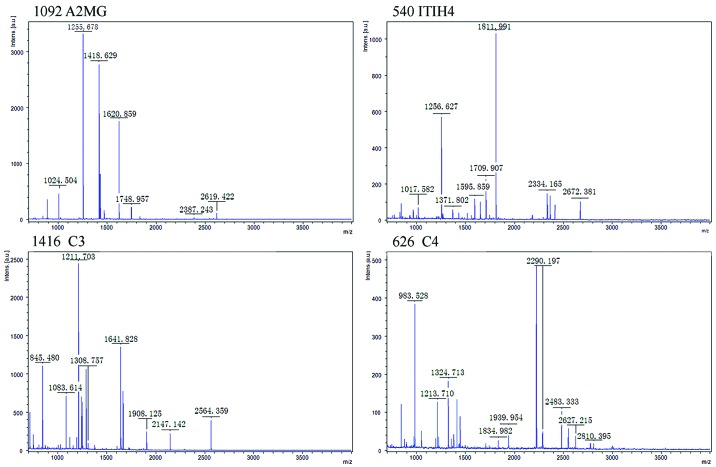
Matrix-assisted laser desorption ionization time-of-flight mass spectrum of each spot. ITIH4, inter-α-trypsin inhibitor heavy chain H4; A2MG, α-2-macroglobulin; C3, complement component 3.

The above four differential protein spots were excised and digested with trypsin in gel for MALDI-TOF peptide mass fingerprinting (PMF) analysis. The product spectra generated by MALDI-TOF-MS/MS were searched against the Swiss-Prot database for exact matches using the MASCOT by PMF (Fig. 2). The four proteins were respectively inter-α-trypsin inhibitor heavy chain H4 (ITIH4), complement component 4 (C4), A2MG and C3 (Table I).

### Western blot analysis and ELISA to confirm the differential expression

**Figure 3:**
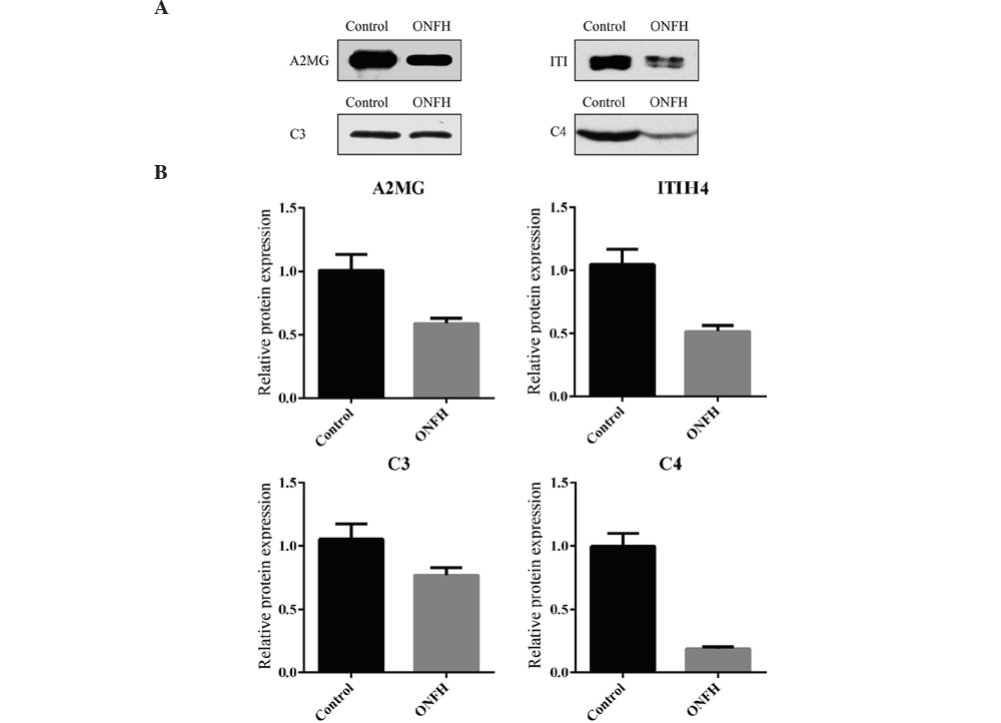
(A) Western blot analysis confirms difference gel electrophoresis findings of decreased protein levels in serum from patients with SONFH. (B) levels of C4, ITIH4, A2MG and C3 are significantly lower in the patient group than in the control group. The control group comprised volunteers and the ONFH group comprised patients with SONFH. SONFH, steroid-induced osteonecrosis of the femoral head; ITIH4, inter-α-trypsin inhibitor heavy chain H4; A2MG, α-2-macroglobulin; C3, complement component 3.

**Figure 4:**
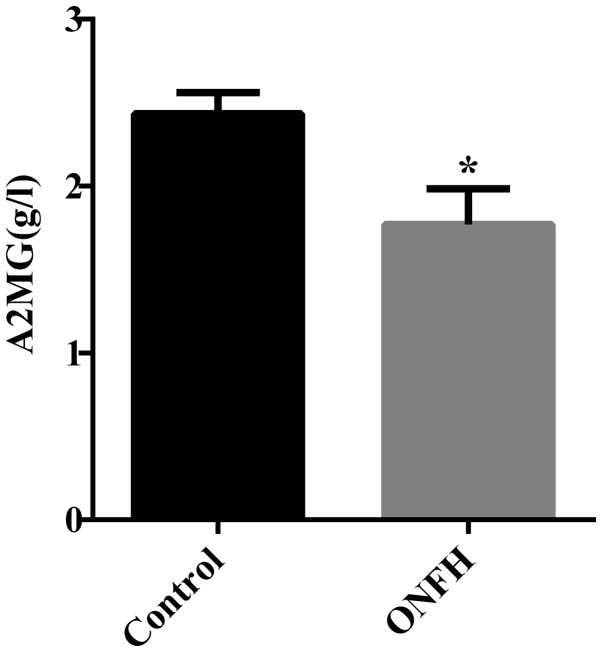
Estimation of A2MG in necrotic bones. A2MG expression was lower in the necrotic bones of patients with SONFH (ONFH) than that in the bones of subjects without SONFH (control). ^*^P<0.05. SONFH, steroid-induced osteonecrosis of the femoral head; A2MG, α-2-macroglobulin.

To validate the differential expressions of these four proteins, the eight serum samples used for the DIGE experiment were assessed by western blot analysis with the specific antibodies. The levels of C4, ITIH4, A2MG and C3 are significantly lower in the patient group than in the control group (Fig. 3), which is consistent with the results from the proteomic study. Subsequently, it was determined whether proteins express differentially during femoral head tissue necrosis. To test if it is the case for A2MG, the proteins prepared from the necrotic femoral head tissue were analyzed by ELISA. The expression of A2MG was significantly lower in patient group than in the control group (Fig. 4), which is consistent with the result of the serum samples. No change was observed in the levels of C3, C4 and ITIH4 in necrotic bone tissues (data not shown).

## Discussion

The incidence of SONFH is increasing year by year, while the diagnosis of this disorder still relies on image examination, which often fails to detect the lesion at the early stage. Therefore, a number of patients miss the opportunities for early treatment. It is important to find the diagnostic biomarkers for SONFH. Since blood tests are commonly used in clinical practice, the protein profile in the serum from the patients with SONFH and healthy volunteers were analyzed using cutting-edge proteomic technology. The aim was to identify proteins whose level in the blood was significantly altered in patients with SONFH. To ensure the reproducibility, accuracy and objectivity of the experiments, the following steps were implemented to minimize the experimental errors between samples. First, the sample selection criteria were strictly followed. Secondly, high abundance proteins were removed prior to the electrophoresis, which otherwise would mask the low abundance proteins. Further, fluorescence labeled 2-D DIGE was employed. An internal standard was used for each protein spot in 2-D DIGE, and the software designed for 2-D DIGE automatically corrected the protein amount according to the internal standard. Thus, these approaches significantly improved the reproducibility and the sensitivity.

In the present study, four proteins (C3, C4, ITIH4 and A2MG) showed lower expression in the serum of patients with SONFH than that of the normal subjects. The changes were confirmed by western blotting. The expressions of these proteins were also examined in necrotic bone tissues. Unlike serum, necrotic bone tissue did not have detectable amounts of C4 and ITIH4. In addition, C3 showed no difference in abundance between the two groups. Only A2MG was downregulated in the protein levels in necrotic bone tissue, consistent with the result of the serum.

The pathogenesis of SONFH remains unclear. Several mechanisms have been proposed, including lipid metabolism dysfunction, intravascular coagulation, apoptosis and reactive oxygen imbalances. The present study shows that the expression of C3, C4, ITIH4 and A2MG were significantly altered in patients with SONFH. All four proteins are closely associated with apoptosis, and therefore the present study supports that apoptosis plays a major role in SONFH.

C3, C4 and their degradation products are cytokines and acute phase reactive proteins that are produced by macrophages and hepatocytes. They are key factors in the activation of the complement system. The activation of the complement cascade can cause a variety of biological effects, including immune response, generation of sensitized lymphocytes and altered metabolism of blood sugar and lipid (13). However, limited studies have systematically elucidated the mechanism by which the immune system affects SONFH. Wu *et al* (14) showed that the complement factor C3 precursor is elevated in the serum of patients with ONFH. This previous study showed that complement factor C3 precursor plays an important role in the homeostasis of inflammation, necrosis or apoptosis in ONFH. The present results show that complement activation is reduced in patients with SONFH. This may be attributed to the immunosuppressive effect of steroids. Excess steroids can suppress complement activation and immune complex formation (15). Familian *et al* (16) found that plasma levels of C3 and C4 increased in the majority of patients with rheumatoid arthritis prior to therapy, but significantly decreased following the start of infliximab (an immunosuppressive agent) treatment. The mechanism of complement inhibition involved in SONFH requires further study.

*ITIH4*, when translated, is secreted into the blood, where it is cleaved by plasma kallikrein into two smaller forms. *ITIH4* mRNA is specifically expressed in the liver. The gene is part of a cluster of similar genes on chromosome 3. Two transcription variants encoding different isoforms have been found. ITIH4 is also an acute phase reactive protein, but its biological function remains unknown. It was detected in swine, bovine and rat models with experimentally-induced acute inflammation (17–19). Pineiro *et al* (20) showed that in humans, *ITIH4* mRNA and the secreted protein are highly upregulated by IL-6 in HepG2 hepatoma cells. Bost *et al* (21) assumed ITIH4 may interact with components of the extracellular matrix and modulate cell migration and proliferation during the development of the acute-phase response. It is clear that ONFH is accompanied by inflammation. Aseptic inflammation presents in patients with ONFH and it is conceivable that persistent consumptive inflammation and the effects of steroids lead to the decrease of serum ITIH4. Further study is necessary to address the role of ITIH4 in the disease.

A2MG is an inhibitor of matrix metalloproteases (MMP) (22), which is mainly synthesized by hepatocytes in the liver. Small amounts of A2MG are also produced by a number of other cells, including lung fibroblasts, macrophages, astrocytes and tumor cells (23,24). A2MG functions as a broad irreversible proteinase inhibitor and is involved in various physiological processes (25,26). A2MG regulates several key factors of SONFH. The conformational change can activate A2MG, resulting in exposure of binding sites for its cell surface receptor, including the low-density lipoprotein receptor-related protein. Upon binding A2MG-proteinase complexes from the extracellular matrix are rapidly removed, which blocks lipid catabolism (27). A2MG modulates blood coagulation. As reported by Simpson *et al* (28), A2MG significantly enhanced plasmin generation. However, A2MG binds vascular endothelial growth factor and the resultant A2MG-complex inhibits heparin activity, leading to elevated coagulation. Human A2MG has been verified to effectively decrease the release of superoxide radicals by polynuclear leukocytes following radiation. The activity of superoxide dismutase in red cells can also be increased. The free radicals and MMP imbalance exist in the pathological process of SONFH. Kerachian *et al* (29) demonstrated that the *A2MG* gene is significantly upregulated in avascular necrosis of the rat femoral head induced with steroids. Along with those findings, the present study showed that A2MG was significantly lower in the bone tissue of patients with SONFH. Lower A2MG may affect the process of SONFH through these aspects. Consistent with the bone tissue, the serum A2MG level was also decreased.

In conclusion, A2MG is involved in multiple mechanisms underlying SONFH, including blood coagulation, hyperlipidemia, free radicals and MMP degradation. This underscores the critical role of A2GM in the development of SONFH. Therefore, A2GM may become a novel potential biomarker and a novel therapeutic target for SONFH.

## Figures and Tables

**Table I:** Proteins presenting significant differences in abundance in the steroid-induced femoral head osteonecrosis group versus the control group.

Position	Master number	T-test	Average ratio	Mw	Identification
1	540	0.024	−1.49	89	ITIH4_HUMAN
2	626	0.048	−2.04	85	CO4A_HUMAN or CO4B_HUMAN
3	1092	0.032	−1.58	67	A2MG_HUMAN
4	1416	0.011	−2.16	48	CO3_HUMAN

Differentially-expressed protein spots were identified from the two-dimensional difference gel electrophoresis profiling of human plasma, with a lower abundance in the steroid-induced femoral head osteonecrosis group compared to the control group. Mw, molecular weight.

## Abbreviations

SONFH: Steroid-induced osteonecrosis of the femoral head
ONFH: osteonecrosis of the femoral head
ITIH4: Inter-α-trypsin inhibitor heavy chain H4
A2MG: α-2-macroglobulin
